# Neutrophil Priming by Cytokines in Patients With Obstructive Jaundice

**DOI:** 10.1155/1994/74202

**Published:** 1994

**Authors:** W. G. Jiang, M. C. A. Puntis, M. B. Hallett

**Affiliations:** University Department of Surgery University of Wales College of Medicine Cardiff UK

## Abstract

Patients with obstructive jaundice frequently suffer postoperative complications. We have investigated the relationship of obstructive jaundice to the neutrophil oxidase response and the “priming” of the response by the cytokines TNF*α*, interleukin-1 (IL-1), IL-6, and IL-8. On stimulation with f-met-leuphe (fmlp), the respiratory burst in neutrophils from jaundiced patients was greatly increased compared with controls (*p* < 0.01), jaundiced patients having the highest respiratory burst levels were those with the poorest prognosis. Neutrophils from controls were primed by all the cytokines tested, whereas “jaundiced” cells were primed only by IL-1, and not by TNF*α*, IL-6, or IL-8, which in fact produced slight inhibition. We conclude that neutrophils from obstructive jaundiced patients have raised oxidative responses which may be due to “pre-priming” *in vivo* by cytokines, such as IL-6, IL-8, or TNF*α*. This exaggeration of the oxidative response in circulating neutrophils may contribute to the peri-operate complications of patients with obstructive jaundice.


*HPB Surgery*, 1994, Vol. 7, pp. 281–289
Reprints available directly from the publisher
Photocopying permitted by license only

© 1994 Harwood Academic Publishers GmbH
Printed in the United States of America

### NEUTROPHIL PRIMING BY CYTOKINES IN PATIENTS WITH OBSTRUCTIVE JAUNDICE

W.G. JIANG, M.C.A. PUNTIS and M.B. HALLETT
*University Department of Surgery, University of Wales College of Medicine, Cardiff, UK*

*(Received 18 February 1993)*

Patients with obstructive jaundice frequently suffer postoperative complications. We have investigated the relationship of obstructive jaundice to the neutrophil oxidase response and the "priming" of the response by the cytokines TNF$\alpha$, interleukin-1 (IL-1), IL-6, and IL-8. On stimulation with f-met-leu-phe (fmlp), the respiratory burst in neutrophils from jaundiced patients was greatly increased compared with controls ($p < 0.01$), jaundiced patients having the highest respiratory burst levels were those with the poorest prognosis. Neutrophils from controls were primed by all the cytokines tested, whereas "jaundiced" cells were primed only by IL-1, and not by TNF$\alpha$, IL-6, or IL-8, which in fact produced slight inhibition. We conclude that neutrophils from obstructive jaundiced patients have raised oxidative responses which may be due to "pre-priming" *in vivo* by cytokines, such as IL-6, IL-8, or TNF$\alpha$. This exaggeration of the oxidative response in circulating neutrophils may contribute to the peri-operate complications of patients with obstructive jaundice.

KEY WORDS: Neutrophils, respiratory burst, obstructive jaundice, tumor necrosis factor, Interleukin-1, Interleukin-6, Interleukin-8

Abbreviations: Fmlp: f-met-leu-phe, GM-CSF: granulocyte-macrophage colony-stimulating factor, IFN: interferon, IL: interleukin, TGF$\beta$: transforming growth factor beta, TNF: tumor necrosis factor, HU r TNF$\alpha$: Human recombinant tumor necrosis factor alpha

### INTRODUCTION

Neutrophils are the largest population of immune cells in the body and play a key role in combating bacterial infections. There is however increasing evidence that, apart from killing pathogens, neutrophils may also cause tissue damage in some pathological conditions such as trauma[1], sepsis[2], adult respiratory distress syndrome (ARDS)[3], and rheumatoid arthritis[4]. Neutrophils secrete toxic products, such as hydrolytic enzymes and toxic metabolites of oxygen, which have the potential to cause damage to neighbouring cells and extracellular matrix. Recently, a number of cytokines have been shown to cause "priming" of neutrophils; a state in which the neutrophils are "prepared" to act upon a secondary stimulus. Treatment of neutrophils with some cytokines, including tumor necrosis factor alpha (TNF$\alpha$)[5,6], interleukin-1 (IL-1)[7], interleukin-6 (IL-6)[8] interleukin-8 (IL-8)[9,10], granulocyte-macrophage colony-stimulating factor (GM-CSF)[11], transforming growth factor-beta (TGF-$\beta$)[12] or interferon-gamma (IFN$\gamma$)[13] causes an increased respiratory burst on stimulation with such agents as the peptide, f-met-leu-phe (fmlp). Since the circulating levels of these cytokines are increased in critically ill patients and as they can increase the neutrophil oxidative responses, they may exacerbate neutrophil-induced tissue damage[1,2,14,15] in certain patients.

Obstructive jaundice patients frequently suffer postoperative complications, such as liver and renal dysfunction[16,17]. It has been shown that these patients have increased levels of circulating endotoxin, a potent stimulant of monocyte cytokine production[18,19]. Furthermore, peripheral blood monocytes from these patients have an increased capacity to produce tumor necrosis factor and interleukin-6[20].

The aims of this study were therefore to establish, in jaundiced patients, the relationship between the neutrophils' oxidative response, their sensitivity to "priming" by cytokines, TNF$\alpha$, IL-1, IL-6, IL-8, and GM-CSF and the immediate clinical outcome of the patient. We report here that neutrophils from jaundiced patients had a greatly increased oxidative response. We suggest that this may be due to cytokine pre-priming of the neutrophils *in vivo*, as we also demonstrate that IL-6, IL-8 and TNF$\alpha$ had no further priming effect on these cells.

### PATIENTS AND MATERIALS

Twenty two patients with obstructive jaundice were included in this study with a mean age of 65.3 $\pm$ 4 years. The cause of the benign biliary obstruction was gallstones ($n$ = 8) or benign biliary stricture ($n$ = 2), and of the malignant obstruction cholangiocarcinoma ($n$ = 8) or pancreatic cancer ($n$ = 5). The control group ($n$ = 18) included normal volunteers, non-jaundiced patients for elective surgery (hernia repair, cholecystectomy, or colectomy for cancer) with a mean age of 54.6 $\pm$ 7 years. All the patients gave informed consent and no patients showed evidence of sepsis. In controls, the white cell count was 6.9 $\pm$ 0.4 $\times$ $10^6$/ml, with neutrophils 4.4 $\pm$ 0.4 $\times$ $10^6$/ml; and in the jaundiced group the white count was 8.0 $\pm$ 0.5 $\times$ $16^6$/ml with neutrophils 6.0 $\pm$ 0.5 $\times$ $10^6$/ml, no patients were pyrexial.

Blood was taken before any other clinical intervention. Peripheral blood was heparinised and processed within one hour. All stages of the separation were carried out at 4° C to avoid activation of the cells. Plasma were kept for bile salt assay (by a commercial kit: "Enzabile", from Nycomed, Norway). Neutrophils were separated by dextran sedimentation, Ficoll-Hypaque separation and a hypotonic technique to remove the contaminating erythrocytes. The isolated cells were 93–95% neutrophils with less than 2% monocyte contamination as determined by non-specific esterase staining. The neutrophils were suspended in HEPES-buffered Krebs medium (NaCl 120mM, KCl 4.8 mM, $Mg_2SO_4$ 1.2 mM, $K_2HPO_4$ 1.2 mM, $CaCl_2$ 1.3 mM, HEPES 25 mM, with 0.1% bovine serum albumin, pH 7.4) and adjusted to $10^7$ cell/ml for experiments.

The neutrophil respiratory burst was monitored using luminol-dependent chemiluminescence as previously described[21]. Briefly, neutrophils were preincubated in the presence of luminol with or without cytokine for 10 minutes in a water bath (37° C) and were then put into the luminometer to record their base line response. Cells were then stimulated with fmlp (1$\mu$M) in the presence of cytochalasin B (5$\mu$g/ml) and the respiratory burst was recorded. Neutrophil oxidative responses are shown as luminescent counts per second (CPS) and the extent of priming calculated from the following formula:

$$\text{Priming Index (PI)} = \frac{\text{CPS with cytokine} - \text{CPS without cytokine}}{\text{CPS without cytokine}} \times 100$$

Human recombinant tumor necrosis factor alpha (Hu rTNF$\alpha$) was kindly provided by Dr N Matthews (Dept of Microbiology, UWCM), 1 ng being equal to 40 international units (the standard used was 87/650, NBSB, England) as assayed by a standardised L929 bioassay[22]. Hu rTNF$\alpha$ was used at 100 pg/ml final concentration. Recombinant GM-CSF (purchased from R&D Systems, Oxford, England) was used at 5.0 ng/ml. Recombinant human IL-1$\beta$ (86/680) and IL-8 (89/520) were kindly provided by NBSB, and were used at final concentrations of 100 U/ml and 1ng/ml respectively. Recombinant human IL-6 was a gift from Dr Aarden, Amsterdam, Netherlands) and was used at 200 U/ml. All other materials were purchased from Sigma (UK).

Student *t*-test was used for statistical analysis and $p < 0.05$ taken as significant. Data are shown as mean ± SEM.

### RESULTS

### *1. Respiratory Burst of Neutrophils from Jaundiced Patients*

The respiratory burst of neutrophils from jaundiced patients stimulated with fmlp was 1532.4 ± 301.8 CPS which was significantly higher than 643.4 ± 117.4 CPS obtained with the control group ($p < 0.01$) (Figure 1). The three highest responses were from patients who died within 7 days of the test being performed. The mean response of these three was massively increased compared with the rest of the jaundiced patients (2154.9 ± 309.6 CPS compared with 1137.6 ± 141.0 CPS, $p < 0.05$).

There was no statistically significant difference between the basal oxidative activity of neutrophils from control or from jaundiced patients (9.4 ± 2.2 CPS and 12.8 ± 4.5 CPS respectively).

### *2. Priming of Jaundiced Neutrophils by Cytokines*

Incubation of neutrophils from controls with Hu rTNF$\alpha$ for 10 minutes, resulted in an increased respiratory burst in response to fmlp, the mean priming index being 54%. However neutrophils from jaundiced patients failed to be primed by the same treatment with Hu rTNF$\alpha$, rather Hu rTNF$\alpha$ caused a slight inhibition of the response, with the priming index being -18% (Figure 2). Similarly, although IL-8 (1ng/ml) and IL-6 (200 U/ml) primed neutrophils from controls (priming index = 53% and 20.0% respectively), the priming of neutrophils from obstructive jaundice was reduced (priming index = -25% and 9% respectively. (Figures 3 and 4).

In contrast, interleukin-1 had a significant priming effect on neutrophil oxidative responses both from controls, priming index = 35%, and from patients with obstructive jaundice, priming index = 15%; (Figure 5). The weak primer GM-CSF (5ng/ml) primed to a lesser extent both the normal neutrophil respiratory burst, priming index = 17% (6 individuals tested) and the response of neutrophils from two jaundiced patients where the priming index was 12.5%.

### *3. Neutrophil Responses and Biochemical Parameters*

In the jaundiced group, the mean plasma bilirubin level was 151.8 ± 31 $\mu$M/l, mean

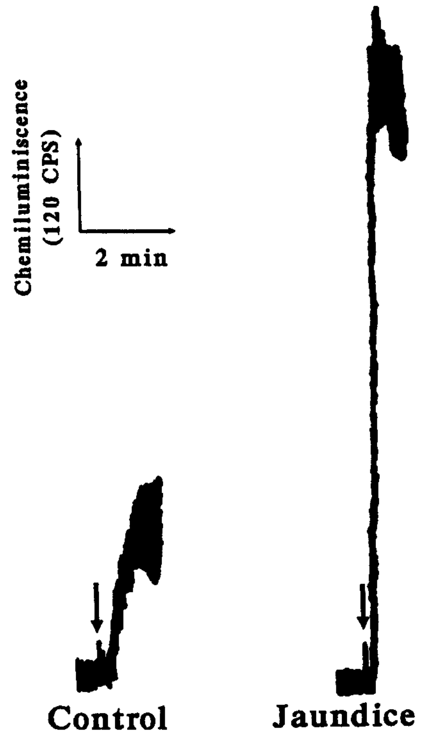

**Figure 1** Neutrophil oxidative responses from a typical control and a jaundiced patient (arrow indicates where fmlp was added). (a): neutrophils from a control showing the base line and respiratory burst. (b): neutrophils from a jaundiced patient, the respiratory burst is much higher than occurs in controls.

albumin level 34.8 ± 1.9 g/l, mean alkaline phosphatase 708 ± 192 $\mu$M/l, and the mean aspartate aminotransferase 87.9 ± 19.7 $\mu$M/l. Bile salt levels in the control and jaundiced groups were 15.4 ± 3.0 $\mu$M/l and 68.9 ± 14.5 $\mu$M/l respectively. The neutrophil base line response showed a minimal correlation with the plasma bile salt levels, r = 0.41, $p < 0.05$, but the respiratory burst, ($r = 0.33, p > 0.05$) did not correlate. Other parameters (bilirubin and, enzyme levels) failed to show any correlation with neutrophil responses.

### DISCUSSION

Although neutrophils are important in combating bacterial infection, their over-activation has been shown to be harmful to the host. This overactivation of neutrophils may have a role in causing damage to the host in sepsis, trauma, ARDS, and rheumatoid arthritis. From the results presented in this paper, we suggest that neutrophil priming and activation may also play a key part in the effect of obstructive jaundice on the host. We demonstrate here that the oxidative

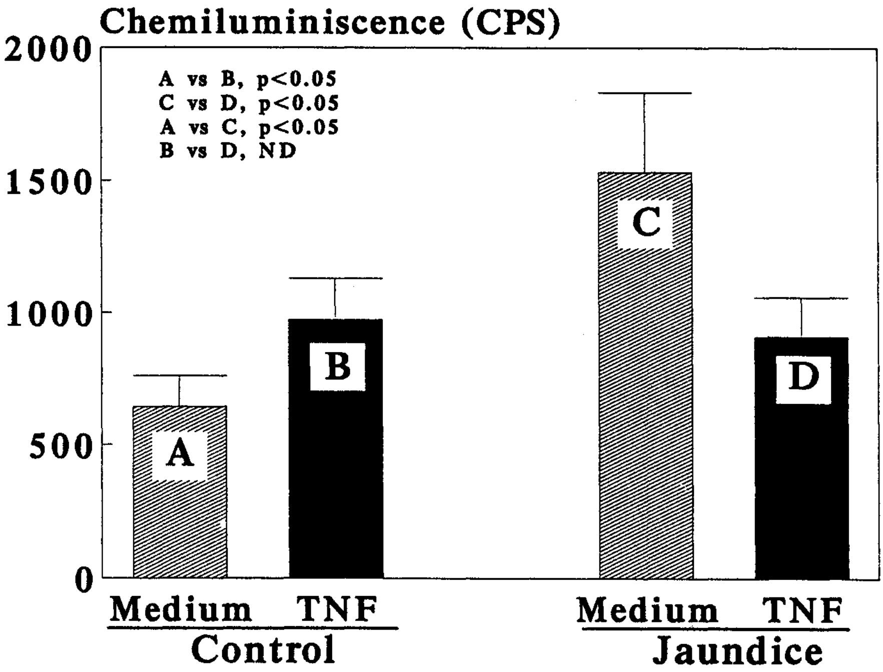

**Figure 2** Neutrophil priming by Hu rTNF. Cells were cultured with TNF (100pg/ml) for 10 minutes and then stimulated with fmlp in the presence of cytochalasin B. Neutrophils from controls (left) an r primed by TNF, but cells from jaundiced patients are inhibited by TNF. Bars indicate mean ± sem.

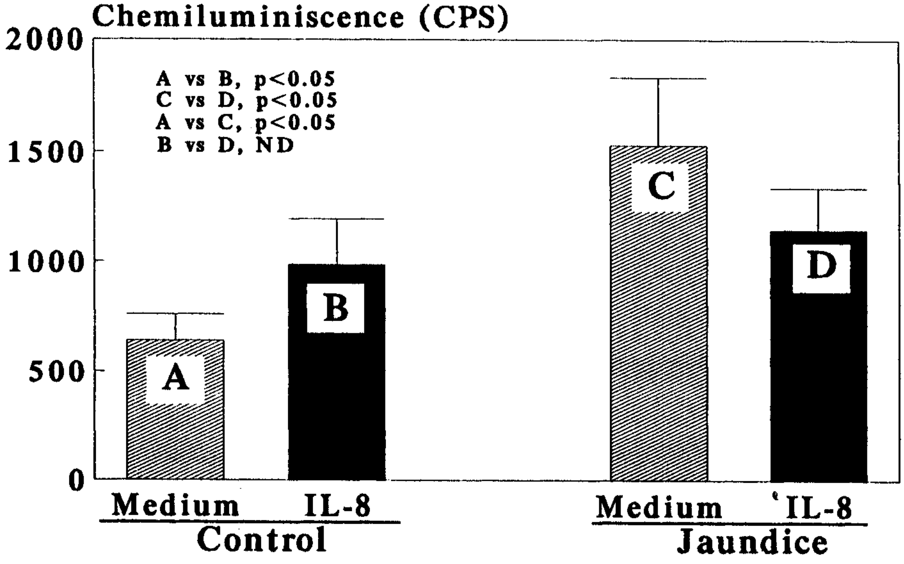

**Figure 3** Neutrophil priming by interleukin-8 at 1.0 ng/ml. Significant priming by IL-8 was observed with control neutrophils. IL-8 has inhibitory effects on the cells from jaundiced patients. Bars indicate mean ± sem.

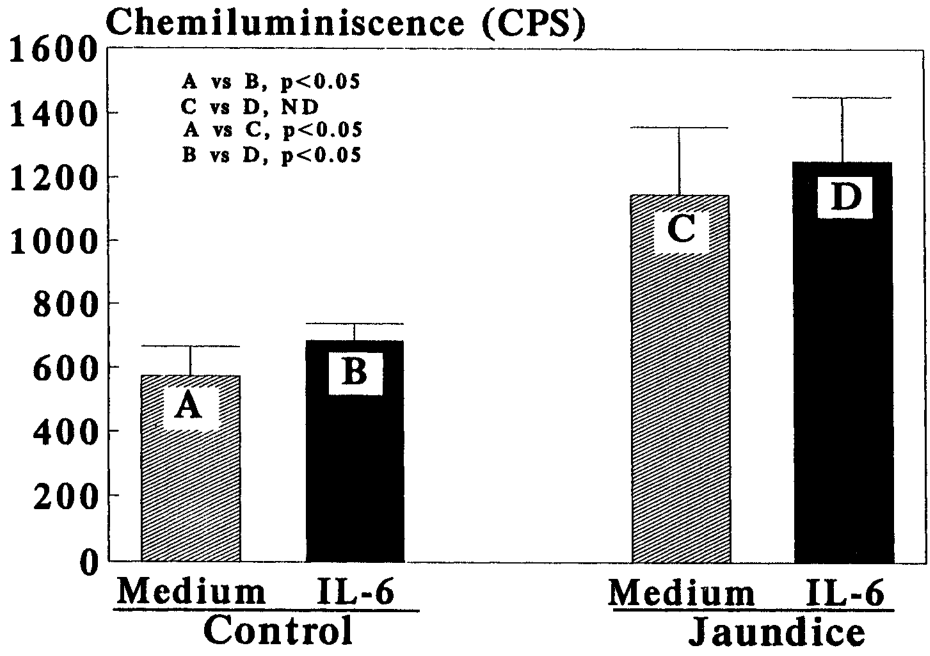

**Figure 4** Neutrophil priming by interleukin-6 at 200 U/ml. IL-6 primes normal neutrophils, but cells from jaundiced patients are less responsive. Bars indicate mean ± sem.

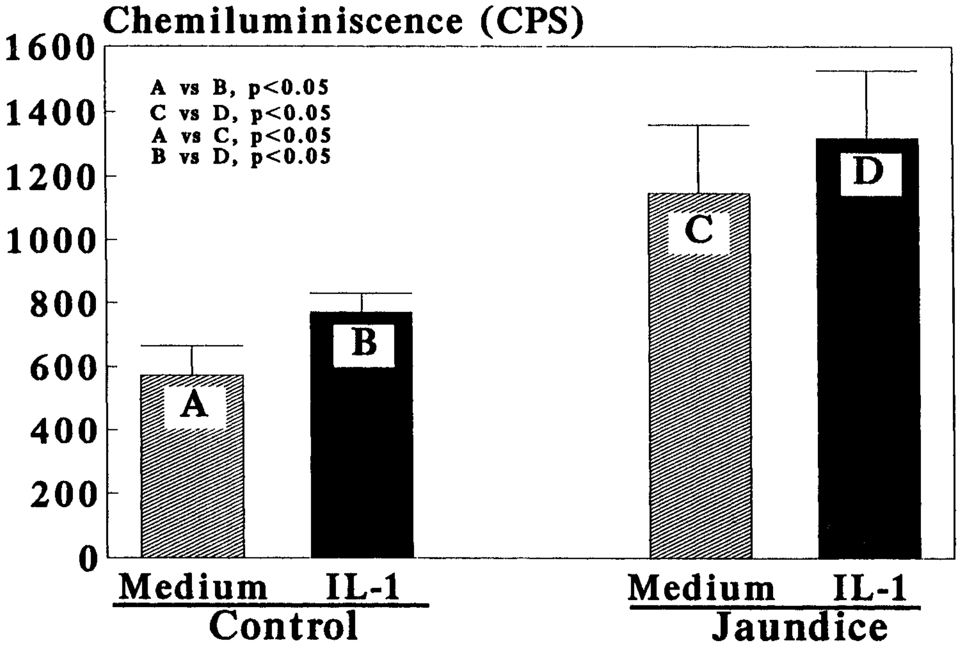

**Figure 5** Neutrophil priming by interleukin-1$\beta$ at 100 U/ml. Cells from both the control and the jaundiced group are primed by IL-1. Bars indicate mean ± sem.

response of neutrophils from jaundiced patients is massively increased. Although the group studied was not large enough to include a large number of critically ill patients, the three patients who died shortly after testing showed an extremely high level of oxidative response. It is not clear which is cause and effect in this situation but this observation clearly warrants further study.

A number of neutrophil primers have been reported recently. Interleukin-1, IL-6, IL-8, TNF$\alpha$, TGF$\beta$, IFN$\gamma$, and GM-CSF all have the property of priming the neutrophil oxidative response. We confirm the ability of IL-1, IL-6, IL-8, TNF$\alpha$ and GM-CSF to prime the oxidative response of neutrophils in the control group. We have also demonstrated that IL-8 and TNF$\alpha$ are potent primers whereas, GM-CSF and IL-6 are relatively weak.

Neutrophils from jaundiced patients compared with controls show markedly different responses. Although the oxidative responses were larger than in controls the effect of the primers especially to IL-6, IL-8 and TNF$\alpha$ was small or nonexistent. These data suggest that neutrophils from jaundiced patients may have been previously exposed to these or other cytokines *in vivo* and are therefore "pre-primed" before the experiment. This suggestion is consistent with previous observations that monocytes from patients with obstructive jaundice produce higher levels of TNF$\alpha$ and IL-6[20] and the circulating levels of cytokines including TGF$\beta$ are also higher in these patients[23]. Bemelmans[24] has reported that mice with obstructive jaundice have increased plasma levels of TNF$\alpha$ and IL-6. The possibility therefore exists that neutrophils from these patients may be primed by the presence of cytokines liberated from activated monocytes *in vivo*, or possibly by the increased circulating endotoxin levels that have been reported in these patients[18,19,25,26]. Indeed, a recent report shows that neutrophil activation is closely related to the circulating level of interleukin-8 in patients with acute pancreatitis[27].

The mechanisms by which prior exposure to cytokines may render neutrophils insensitive to subsequent exposure are not yet fully established. However, activation of neutrophils by some stimuli, including fmlp, results in the "shedding" of TNF$\alpha$ membrane receptors[28,29]. Furthermore, it has been reported that neutrophils from patients with sepsis have reduced cytokine receptor numbers and decreased binding capacities for the cytokines IL-6 and TNF$\alpha$[30]. The possibility therefore exists that activation of neutrophils *in vivo* leads to a reduction in cytokine receptor number or binding capacity, rendering them insensitive to subsequent attempts to further prime these cells with cytokines. Another possibility is that prolonged exposure to cytokines renders them insensitive to their presence. *In vitro* prolonged incubation of normal neutrophils with IL-3, IL-6, IL-8, and TNF has been reported to initially enhance leukotriene release and then to "deactivate" this response[31]. Neutrophils from jaundiced patients may thus be desensitized to certain cytokines by their prolonged pre-exposure in the circulation.

The lack of desensitization to IL-1 and possibly GM-CSF suggests that either these two cytokines are not elevated in jaundiced patients, or that the mechanisms responsible for desensitisation to the other cytokines do not operate for these cytokines. It is interesting to note that in contrast to the monocyte production of the other cytokines, IL-1 production in jaundiced patients remain unchanged[20].

This study has therefore shown that neutrophils from patients with obstructive jaundice have a greatly increased oxidative response, possibly due to prior exposure to circulating cytokines, and that for patients with an especially high oxidative response and desensitization to certain cytokines this might be potentially harmful and effect their immediate prognosis.

*Acknowledgements*

We thank Dr N. Matthews, Dr L.A. Aarden and NBSB (England) for their kind gifts of cytokines. This work is supported by Scotia Pharmaceutical Ltd (UK) and Arthritis and Rheumatism Council (UK).

*References*

1. Tanaka, H., Ogura, H., Yokota, J., Sugimoto, H., Yoshioka, T. and Sugimoto, T. (1991) Acceleration of superoxide production from leukocytes in trauma patients. *Ann. Surg.*, **214**, 187–192
2. Trautinger, F., Hammerle, A.F., Poschl, G. and Micksche, M. (1991) Respiratory burst capability of polymorphonuclear neutrophils and TNF-alpha serum levels in relationship to the development of septic syndrome in critically ill patients. *J. Leukocyte Biol.*, **49**, 449–454
3. Rivkind, A.I., Siegel, J.H., Littleton, M., De Gaetano, A., Mamantov, T., Laghi, F. and Stoklosa, J.C. (1991) Neutrophil oxidative burst activation and the pattern of respiratory physiologic abnormalities in the fulminate post-traumatic adult respiratory distress syndrome. *Circulatory Shock*, **33**, 48–62
4. Nurcombe, H.L., Bucknall, R.C. and Edwards, S.W. (1991) Neutrophils isolated from the synovial fluid of patients with rheumatoid arthritis: priming and activation *in vivo*. *Ann. Rheumat. Dis.*, **50**, 147–153
5. Klebanoff, S.J., Vadas, M.A., Harlan, J.M., Sparks, L.H., Gamble, J.R., Agosti, J.M. and Waltersdorph, A.M. (1986) Stimulation of neutrophils by tumor necrosis factor. *J. Immunol.*, **136**, 4220–4225
6. Berkow, R.L. and Dodson, M.R. (1988) Biochemical mechanisms involved in the priming of neutrophils by tumor necrosis factor. *J. Leukocyte Biol.*, **44**, 345–352
7. Sample, A.K. and Czuprynski, C.J. (1991) Priming and stimulation of bovine neutrophils by human interleukin-1 alpha and tumor necrosis factor alpha. *J. Leukocyte Biol.*, **49**, 107–115
8. Borish, L., Rosenbaum, R., Albury, L. and Clark, S. (1989) Activation of neutrophils by recombinant interleukin-6. *Cell Immunol.*, **121**, 280–289
9. Djeu, J.T., Matrushima, K., Oppenheim, J.J., Shiotruki, K. and Blanchard, D.K. (1990) Functional activation of human neutrophils by recombinant monocyte derived neutrophil chemotactic factor / IL-8. *J. Immunol.*, **144**, 2205–2210
10. Baggiolini, M., Walz, A. and Kunkel, S.L. (1989) Neutrophil-activating peptide-1/interleukin-8, a novel cytokine that activates neutrophils. *J. Clin. Invest.*, **84**, 1045–1049
11. Mege, J.L., Gomez-Cambronero, J., Molski, T.F.P., Becker, E.L. and Sha'afi, R.I. (1989) Effect of granulocyte-macrophage colony-stimulating factor on superoxide production in cytoplasts and intact human neutrophils. *J. Leukocyte Biol.*, **46**, 161–168
12. Brandes, M.E., Mai, U.E., Ohura, K. and Wahl, S.M. (1991) Type I transforming growth factor-beta receptors on neutrophils mediate chemotaxis to transforming growth factor-beta. *J. Immunol.*, **147**, 1600–1606
13. Diamond, R.D., Lyman, C.A. and Wysong, D.R. (1991) Disparate effects of interferon-gamma and tumor necrosis factor-alpha on early neutrophil respiratory burst and fungicidal responses to Candida albicans hyphae *in vitro*. *J. Clin. Invest.*, **87**, 711–720
14. Tredgett, E.E., Yu, Y.M., Zhong, S., Burini, R., Okusawa, S., Gelfand, J.A., Dinarello, C.A., Young, V.R. and Burke, J.F. (1988) Role of interleukin-1 and tumor necrosis factor on energy metabolism in rabbits. *Am. J. Physiol.*, **255**, E760–768
15. Mozes, T., Ben-Efraim, S., Tak, C.J.A.M., Heiligers, J.P.C., Saxena, P.R. and Bonta, I.L. (1991) Serum levels of tumor necrosis factor determine fatal or non-fatal course of endotoxic shock. *Immunol. Lett.*, **27**, 157–162
16. Pain, J.A., Cahill, C.J. and Bailey, M.E. (1985) Perioperative complications in obstructive jaundice: therapeutic considerations. *Br. J. Surg.*, **72**, 942–945
17. Wait, R.B. and Kahng, K.V. (1989) Renal failure complicating obstructive jaundice. *Am. J. Surg.*, **157**, 256–263
18. Hunt, D.R., Allison, M.E.M., Prentice, C.R.M. and Blumgart, L.H. (1982) Endotoxemia disturbance of coagulation and obstructive jaundice. *Am. J. Surg.*, **144**, 325–329
19. Wardle, E.N. and Wright, N.A. (1970) Endotoxin and acute renal failure associated with obstructive jaundice. *Br. Med. J.*, **4**, 472–474

20. M.C.A. Puntis and W.G. Jiang (1992) Monocyte from obstructive jaundice patients show increased TNF and IL-6 production. *Br. J. Surg.*, **79**, 458
21. Hallett, M.B., Luzio, J.P. and Campbell, A.K. (1981) Stimulation of $Ca^{2+}$ dependent chemiluminiscence in rat polymorphonuclear leukocytes by polystyrene beads and the non-lytic action of complement. *Immunol.*, **44**, 569–576
22. Neale, M.L., Williams, B.D. and Matthews, N. (1989) Tumour necrosis factor activity in joint fluids from rheumatoid patients. *Br. J. Rheumatol.*, **28**, 104–108
23. M.C.A. Puntis and W.G. Jiang (1992) Monocyte and plasma levels of transforming growth factor beta in patients with obstructive jaundice. *HPB Surg.*, **5** (Suppl), 6
24. Bememans, M.H.A., Gouma, D.J., Greve, J.W. and Buurman, W.A. (1992) Cytokines tumor-necrosis factor and interleukin-6 in experimental biliary obstruction in mice. *Hepatol.*, **15**, 1132–1136
25. Guthrie, L.A., McPhail, L.C., Henson, P.M. and Johnson, R.B. (1984) Priming of neutrophils for enhanced release of oxygen metabolites by bacterial lipopolysaccharide. *J. Exp. Med.*, **160**, 1656–1671
26. Aida, Y. and Pabst, M.J. (1991) Neutrophil responses to lipopolysaccharide. Effect of adherence on triggering and priming of the respiratory burst. *J. Immunol.*, **146**, 1271–1276
27. Gross, V., Anderseen, R., Leser, H.G., Ceska, M., Liehl, E., Lausen, M., Fartmann, E.H. and Scholmerich, J. (1992) Interleukin-8 and neutrophil activity in acute pancreatitis. *Eu. J. Clin. Invest.*, **22**, 200–203
28. Porteu, F. and Nathan, C. (1990) Shedding of tumor necrosis factor receptors by activated human neutrophils. *J. Exp. Med.*, **172**, 599–607
29. Johnson, S.E. and Baglioni, C. (1988) Tumor necrosis factor receptors and cytocidal activity are down-regulated by activators of protein kinase C. *J. Biol. Chem.*, **263**, 5686–5692
30. Simms, J. and D'Amice, R. (1992) Intra-abdominal sepsis alters tumor necrosis factor-$\alpha$ and interluekin-1 $\beta$ binding to human neutrophils. *Crit. Care Med.*, **20**, 11–16
31. Brom, J. and Konic, W. (1992) Cytokine-induced (interleukin-3, -6, and -8 and tumor necrosis factor-beta) activation and deactivation of human neutrophils. *Immunol.*, **75**, 281–285

*(Accepted by S. Bengmark 23 April 1993)*

